# Flow analysis determination of iodide at nanogram levels in water

**DOI:** 10.1155/S146392469600020X

**Published:** 1996

**Authors:** C. K. Chandrawanshi, S. K. Chandrawanshi, K. S. Patel

**Affiliations:** School of Studies in Chemistry Pt. Ravishankar Shukla University Raipur MP 492 010 India


Journal of Automatic Chemistry, Vol. 18, No. 5 (September–October 1996), pp. 181–186

### Flow analysis determination of iodide at nanogram levels in water

C. K. Chandrawanshi, S. K. Chandrawanshi and K. S. Patel*

*School of Studies in Chemistry, Pt. Ravishankar Shukla University, Raipur-492 010, MP, India*

*This paper describes a new method for flow injection analysis (FIA) determination of iodide at nanogram levels in water based on the catalytic destruction of the colour of $Fe(III)$-$SCN^-$-$CP^+$ (cetylpyridinium chloride) complex. The apparent molar absorptivity of the complex in terms of iodide is (6·90) × $10^5$ l mole$^{-1}$ cm$^{-1}$ at a maximum absorption of 470 nm. The detection limit of the method is 0·1 ng $I^-$/ml. The FIA variables for the determination of iodide in the water were optimized. Sample throughput was 50 samples/h. The method is particularly useful for characterization of iodide at lower nanogram levels in rain, surface and ground water samples.*

### Introduction

Iodine is a micronutrient which people need in trace levels to synthesize the thyroxine hormone in the thyroid gland. If it is not present in drinking water, serious biochemical disturbances can result, for example, cretinism and myxedema [1]. These diseases are very common in tropical countries such as India where the rainfall is very high. Many methods (for example spectrophotometric, catalytic, ion-selective and ion chromatography) have been reported for the characterization of iodide in water [2–15]. The conventional spectrophotometric methods consume large amounts of reagents and samples with very poor sample analysis rates [2–5]. Iodide is usually determined through its catalytic effect on the Ce(VI)–As(III) system, either by measuring absorption at 405 nm or the fluorescence at 260 and 360 nm. However, this reaction is not very sensitive nor is it particularly selective [6–7]. Flow injection analysis methods based on the catalytic destruction of the colour of the Fe(III)–$SCN^-$ complex are used for monitoring iodide but they have some shortcomings, for example poor sensitivity and low sample throughput [8–10]. The cathodic stripping voltammetric methods for analysis of iodide are the most sensitive (i.e. up to 25 ng/l) but they require a preconcentration of iodide into a static mercury drop or carbon wool at $Ag_3I$ electrode [11–12].

Ion-chromatography is widely used for monitoring iodide, but the sample throughput is low and many separation steps are needed to monitor iodide in the polluted water [13–15]. This paper describes a new, simple, fast, selective, and sensitive flow injection analysis (FIA) method for spectrophotometric determination of total iodide based on the catalytic destruction of the colour of Fe(III)–$SCN^-$–$CP^+$ (where $CP^+$ = cetylpyridinium cation) with $NO_2^-$. The method enhances the sensitivity of the convention catalytic Fe(III)–$SCN^-$ method with better sample throughput.

* *Correspondence to K. S. Patel.*

### Experimental

### *Apparatus*

A Tecator FIA analyser type-5012 equipped with ALPKEM UV-VIS spectrophotometer type-510 (matched with 5·5 mm flow cell) was used for the monitoring work. This system is shown in figure 1.

### *Reagents*

All chemicals used were of analytical grade from E. Merck. A standard solution of iodide (1000 ppm) was prepared by dissolving 1·308 g KI in de-ionized double distilled water and diluted to 1 l. The working solution was prepared by diluting the stock solution. Fresh, degassed and filtered solutions of ferrous ammonium sulphate (0·05 M) in 4·0 M $HNO_3$, sodium nitrite (0·0015 M), and potassium thiocyanate (0·008 M) + cetylpyridinium chloride (CPC) (0·00055 M) were used.

### *Procedure*

The injection time and delay time were fixed at 25 s and 50 s, respectively. The carrier and the reagent solutions were run through silicon tubes by peristaltic pumps. The base line with zero absorbance at absorption maximum 470 nm was recorded at a flow rate 3·2 ml/min. A 200 µl aliquot of the standard solution containing 100 ppb iodide was injected at point S. The colour of the flowing stream was quantitatively decreased to the amount of the iodide injected and the decrease in colour was plotted by setting the plotter at the paper's maximum value.

### Results and discussion

### *Absorption characteristics*

The method's chemistry involved a remarkable colour enhancement of the Fe(III)–$SCN^-$ complex with the surfactant: i.e. cetylpyridinium chloride (CPC). Also the colour of the Fe(III)–$SCN^-$–$CP^+$ complex was removed by $NO_2^-$ in proportion to the amount of the catalyst, i.e. $I^-$ or $IO_3^-$ at between 50 and 60°C. The Fe(III)–$SCN^-$ complex showed a sharp absorption maximum at around 470 nm. The signal peak height in the absence and presence of CPC is shown in figure 2. CPC enhances (up to three-fold) the signal peak height and the synergy, in

0142-0453/96 $12.00 © 1996 Taylor & Francis Ltd.

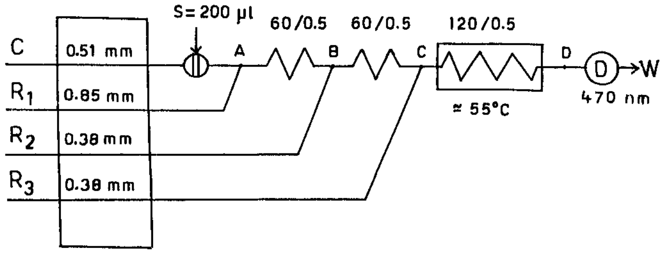

*Figure 1. Schematic diagram of FIA system. Where C = Carrier, deionized double distilled water; $R_1$ = Ferrous ammonium sulphate, 0·05 M in 4·0 M $HNO_3$; $R_2$ = Sodium nitrite, 1·5 × $10^{-3}$ M; and $R_3$ = Mixed reagent solution, 8·0 × $10^{-3}$ M; potassium thiocyanate + 5·5 × $10^{-4}$ M cetylpyridinium chloride.*

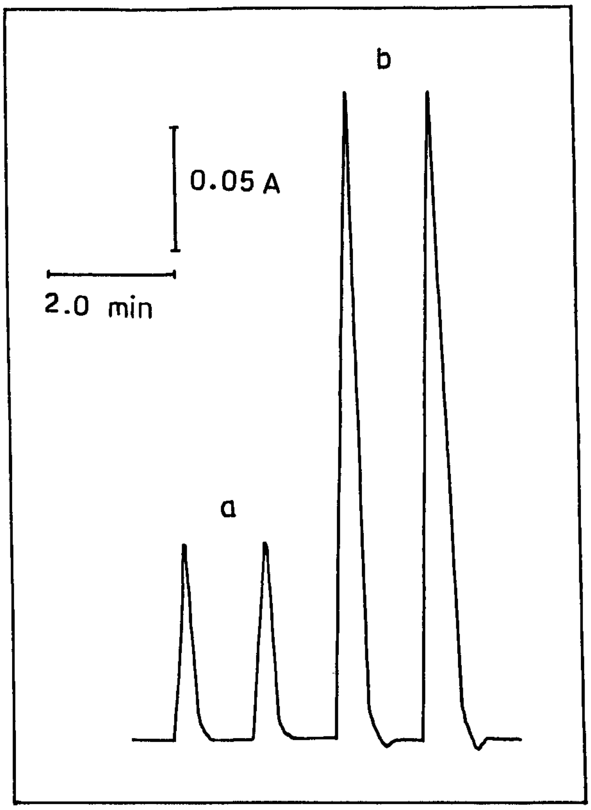

*Figure 2. Hyperchromic shift of the complex in the presence of CPC, 90 ppb iodide at gain factor 2. (a) in the absence of CPC; (b) in the presence of CPC.*

terms of strong hyperchromic shift of the complex, this was found to be 3·25.

### *Effect of acids*

The effects of the acidity of the sample, and reagent solutions on the peak height of the signal were investigated. The maximum peak height of signal was observed when the acidity of the analyte solution, and the reagent solution ($R_1$) was between pH 1–10, and 3·0–4·5 M $HNO_3$, respectively. Beyond 4·5 M $HNO_3$, no smooth base line was recorded which could be due to the oxidation of thiocyanate ions.

### *Effect of reagent concentration*

It was found that optimum concentration range of sodium nitrite, ferrous ammonium sulphate, and potassium thiocynate should be between (0·2–5·0) × $10^{-3}$, (4.0–7·0) × $10^{-2}$, and (4·0–9·0) × $10^{-3}$ M, respectively, to achieve the maximum peak height of signal. Adding more reagents beyond these limits caused problems in getting a smooth base line. The effects of various types of surfactants on the colour enhancement of the Fe(III)–$SCN^-$ complex were investigated, these were: sodium lauryl sulphate, TX-100, TX-300, cetyltrimethylammonium bromide and cetylpyridinium chloride. Of these, only cationic surfactants, cetylpyridinium chloride (CPC) and cetyltrimethylammonium bromide (CTAB), produced a significant increase in the absorbance of the Fe(III)–$SCN^-$ complex (see figure 3). With CTAB, no smooth base line was recorded due to the formation of a slightly soluble yellowish species either with $SCN^-$ or Fe(III)–$SCN^-$ complex or both. Therefore, CPC was selected for the detailed investigation. At least 0·02%, w/v (5·5 × $10^{-4}$ M) CPC was needed to get the maximum peak height and its further addition up to 0·1%, w/v (2·8 × $10^{-3}$ M) had no adverse effect in the signal peak height.

### *Optimum concentration range, sensitivity and statistics*

The present method followed a straight line over the concentration range of 0–100 ppb $I^-$ with slope, intercept, and correlation coefficient of 0·13, −0·35, and +0·99, respectively (see figure 4). The apparent molar absorptivity of the complex in the terms of iodide is (6·90) × $10^5$ l $mole^{-1}$ $cm^{-1}$ at $\lambda_{max}$ 470 nm. The

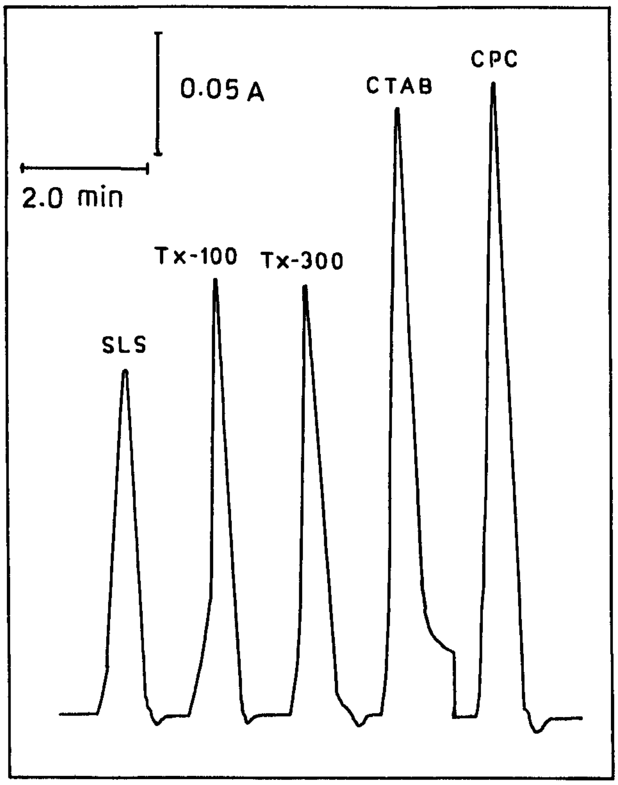

*Figure 3. Effect of various surfactants in the signal peak height, 90 ppb iodide at gain factor 2.*

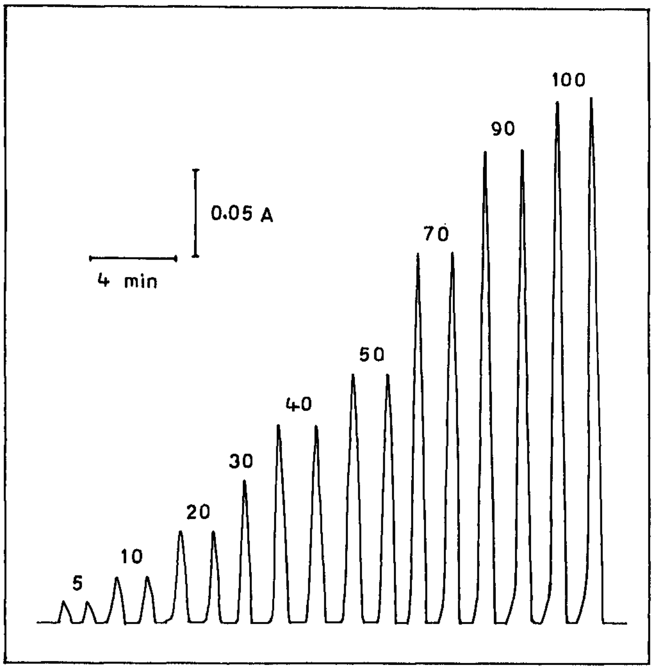

*Figure 4. Signal peak height recorded for standard solution of iodide, 5–100 ppb with the FIA system at gain factor 2.*

detection limit was found to be 0·1 ng $I^-$/ml at gain factor 2. The RSD for six replicate measurements for 80 ng $I^-$/ml at gain factor 2 was found to be $\pm 1{\cdot}2\%$ (see figure 5).

*Effect of diverse ions*

The effect of diverse ions in the analysis of 50 ng $I^-$/ml was examined separately. Different solutions of diverse ions containing 50 ng $I^-$/ml were investigated; none of the diverse ions tested interfered to any significant extent in the determination of iodide (see table 1).

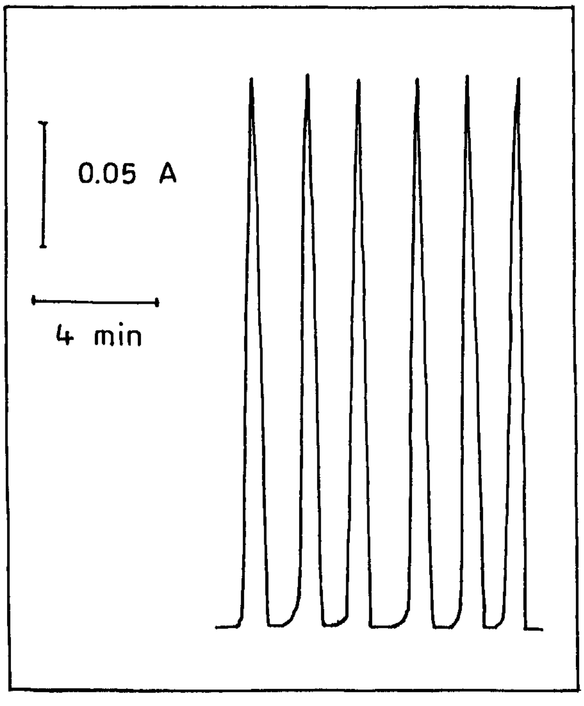

*Figure 5. Signal peak height recorded for standard deviation containing 80 ppb iodide at gain factor 2.*

*Table 1. Effect of diverse ions on determination of 50 ng $I^-$/ml by FIA.*

| Diverse ions | Tolerance limit* µg/ml |
|---|---|
| $PO_4^{3-}$ | 4 |
| Si(IV) | 5 |
| $S^{2-}$ | 6 |
| Cr(VI) | 10 |
| As(V), $F^-$ | 15 |
| Ta(V), Cd(II) | 20 |
| Cu(II), Zn(II), Co(II), Ti(IV) $BO_3^{3-}$, $C_2O_4^{2-}$ | 50 |
| Mn(II), V(V), $Br^-$ | 70 |
| Pb(II), Bi(III) | 100 |
| $HCO_3^-$ | 150 |
| Ca(II) | 200 |
| K(I), $Cl^-$ | 400 |
| Mg(II) | 500 |
| $SO_4^{2-}$ | 2500 |

*Causing an error of less than 2%.

*Effect of bore size, tube length, and other variables*

The effect of bore size of silicon tubes, the length of Teflon tubes, sample volume, rise time, injection time, and delay time were all examined. The maximum peak height of the signal was obtained when the bore size of C, $R_1$, $R_2$, and $R_3$ silicon tubes were 0·51, 0·85, 0·38, and 0·38 mm, respectively. The length of Teflon tube (diameter 0·5 mm) between merging zones A-B, B-C, and C-D needs to be 60, 60, and 120 cm, respectively to obtain maximum peak height. The effect of size of the sample volume injected was studied and a 200 µl aliquot of sample gave the highest signal peak height at flow rate, 3·2 ml/min. Similarly, the effect of rise, injection, and delay time were studied and the maximum peak height was recorded when their values were 1·0, $\geqslant 25$, and $\geqslant 50$ s, respectively. The sample throughput was 50 samples/h.

*Effect of temperature*

The effect of variations in the temperature of the water bath on the rate of destruction of Fe(III)–$SCN^-$–$CP^+$ complex with $NO_2^-$ in the presence of $I^-$ was examined. The optimum temperature of the water bath for the maximum destruction should be between 50°C and 60°C. Below 50°C the rate of destruction of the complex gradually decreases, whereas above 60°C the destruction rate is the same but the rate of dissociation of the thiocyanato complex increases.

### Reaction mechanism

The catalytic effect of iodide or iodate in the destruction of thiocynate ions was proposed by Oguma *et al.* [10]

$$2SCN^- + 3NO_3^- + 3NO_2^- + 2H^+ \rightleftharpoons 2CN^- + 2SO_4^{2-} + 6NO + H_2O$$

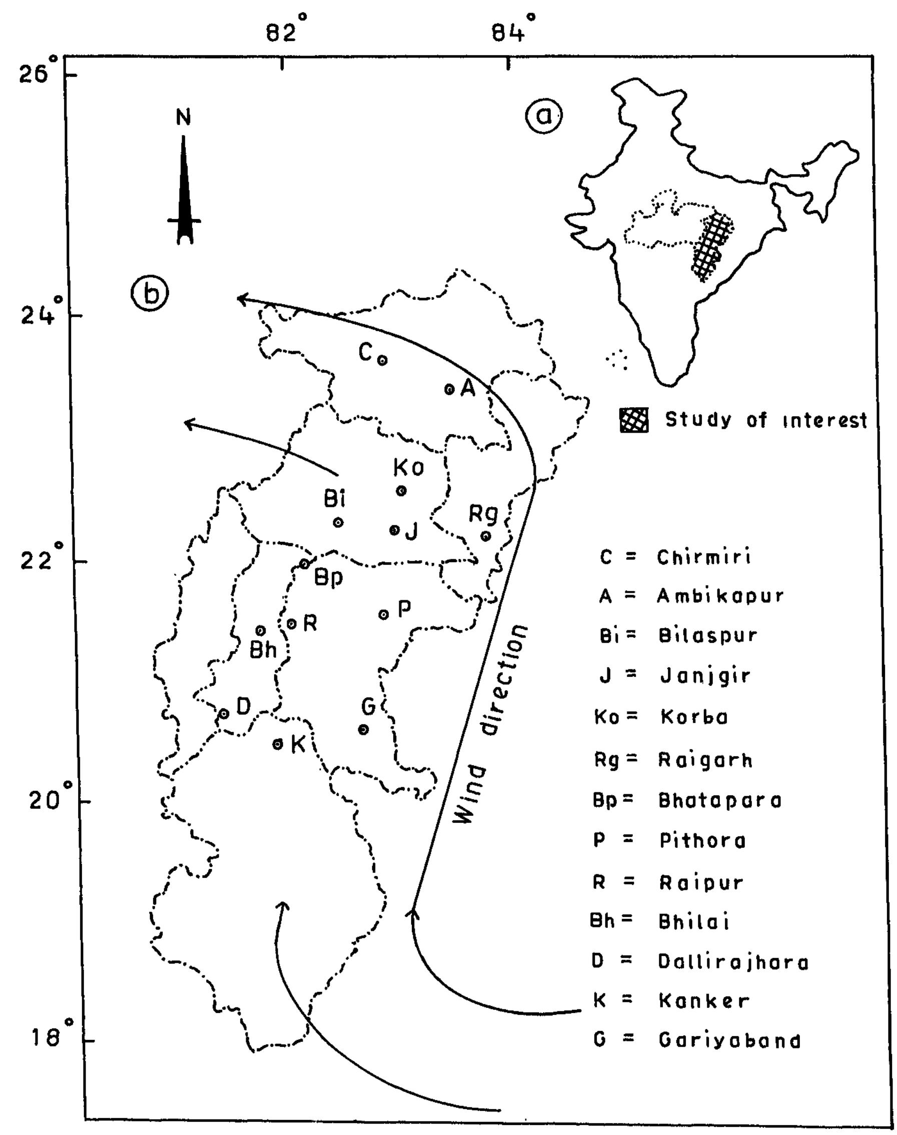

*Figure 6. Geographical map of (a) India; (b) central India.*

In the FIA determination of iron (III) with thiocyanate in the presence of CPC, the composition was determined by the curve-fitting method and found to be 1 : 6 : 2, respectively.

$$Fe^{3+} + 6SCN^- + 2CP^+ \rightleftharpoons \{CP_2[Fe(SCN)_6]\}^-$$

The value of apparent molar absorptivity of this complex was (1·90) × $10^4$ l mole$^{-1}$ cm$^{-1}$ at $\lambda_{max}$ 470 nm [16].

In this work, the value of molar absorptivity of the complex in terms of iodide was found to be (6·90) × $10^5$ l mole$^{-1}$ cm$^{-1}$ at absorption maximum, 470 nm. This value in the terms of iron should be (2·90) × $10^5$ l mole$^{-1}$ cm$^{-1}$.

The ratio of two values: 291 000 and 19 000 is 14·5 close to integer 14. Hence, the expected number of Fe(III)–SCN$^-$–CP$^+$ that can be destroyed by one mole of iodide or iodate should be 14.

$$14\ \{(CP)_2[Fe(SCN)_6]\}^- + 126NO_3^- + 126NO_2^- + 84H^+ \rightleftharpoons 84CN^- + 84SO_4^{2-} + 252NO + 42H_2O$$

*Application of the method*

The validity of the present method was checked with a spectrophotometric method, which was based on oxidation of $I^-$ into $I_2$ with $NaNO_2$, preconcentration of $I_2$ into toluene and reaction of the extract with brilliant green in sequence having detection limit 4 ng $I^-$/ml water [5]; and an ion chromatographic method. The methods were applied for the analysis of total iodide to surface, ground and rain water; the results obtained by these three methods were comparable, see table 2. However, the sample throughput with the solvent extraction and ion chromatography methods was very low. In addition, the precision was low with the ion – chromatography method.

The present method was also used to determine the iodide flux precipitated with rain water in central India during 1995. Rain water was collected at 13 sites (see figure 6) in a plastic bucket on the roofs of the buildings (height at least 13 feet), according to a procedure prescribed by GAW Switzerland. The fluxes precipitated at the 13 sites were in the range of 0·06–5·04 µg/m$^2$/min (see table 3). The high flux of iodide in the monsoon period (July to September) was due to high frequency

*Table 2. Determination of iodide in water.*

| | | Iodide concentration found by | | | | | |
|---|---|---|---|---|---|---|---|
| | | Present method | | Brilliant green | | Ion chromatography | |
| Site | Type of water | ng/ml | ±%RSD | ng/ml | ±%RSD | ng/ml | ±%RSD |
| Raipur | Rain | 1·9 | 1·1 | 2·3 | 1·0 | 2·4 | 2·3 |
| | Surface | 7·2 | 1·0 | 7·6 | 1·2 | 6·9 | 4·3 |
| | Ground | 11·6 | 1·0 | 11·9 | 0·9 | 10·7 | 5·0 |
| Bhilai | Rain | 5·4 | 1·2 | 7·6 | 1·2 | 6·5 | 2·5 |
| | Surface | 7·2 | 1·1 | 7·4 | 1·4 | 6·8 | 4·6 |
| | Ground | 45·9 | 1·0 | 46·1 | 1·5 | 44·2 | 5·3 |
| Korba | Rain | 15·3 | 1·0 | 15·0 | 1·0 | 16·6 | 1·9 |
| | Surface | 0·8 | 1·4 | 1·2 | 1·4 | — | — |
| | Ground | 1·2 | 1·1 | 1·3 | 1·3 | — | — |

*Table 3. Iodide flux precipitated during 1995 in central India.*

| | Iodide flux, μg/m$^2$/min (No. of events) | | | | | | | | | | |
|---|---|---|---|---|---|---|---|---|---|---|---|
| Site | Jan | Feb | March | April | May | June | July | Aug | Sept | Oct | Nov |
| Raipur | 0·12 (1) | 0·18 (2) | 0·36 (4) | 1·02 (2) | 2·40 (6) | 4·44 (8) | 3·36 (14) | 3·78 (12) | 5·04 (6) | 0·66 (2) | 0·48 (1) |
| Korba | — | — | 0·18 (2) | 0·06 (1) | 0·42 (1) | 1·38 (1) | 3·48 (5) | 2·16 (3) | 3·00 (3) | 0·54 (1) | 0·30 (1) |
| Bhilai | — | 0·12 (1) | 0·54 (5) | 1·98 (3) | 3·90 (5) | 0·60 (2) | 4·62 (15) | 4·08 (8) | 0·42 (2) | — | — |
| Bilaspur | — | 0·24 (1) | 0·18 (2) | 0·12 (1) | 1·50 (4) | 2·94 (4) | 3·78 (12) | 3·48 (13) | 1·80 (2) | 1·20 (2) | — |
| Gariyaband | — | 0·12 (1) | 0·36 (3) | 1·50 (4) | 2·52 (3) | 0·78 (4) | 2·28 (10) | 1·74 (3) | — | 1·08 (1) | — |
| Kanker | — | 0·30 (1) | — | — | 1·26 (3) | 2·94 (1) | 3·36 (8) | 2·52 (6) | 1·50 (2) | 0·42 (2) | — |
| Dallirajhara | — | 0·24 (1) | 0·48 (2) | — | 0·24 (1) | 0·60 (2) | 2·76 (7) | 1·32 (5) | — | 0·12 (1) | — |
| Janjgir | — | — | 0·06 (1) | — | 0·18 (1) | 0·42 (2) | 0·84 (3) | 1·86 (6) | 0·30 (2) | 0·60 (1) | 0·78 (1) |
| Raigarh | — | — | — | — | 1·02 (2) | — | 2·10 (4) | 2·40 (5) | 1·80 (4) | 0·78 (2) | 0·42 (1) |
| Chirmiri | — | — | — | — | — | 1·14 (1) | 1·02 (4) | 0·78 (6) | 1·20 (2) | — | — |
| Pithora | — | — | — | — | 1·08 (2) | 0·03 (1) | 0·60 (3) | 0·18 (2) | 2·40 (2) | — | — |
| Bhatapara | — | 0·06 (1) | 0·36 (2) | — | — | 0·42 (1) | 1·38 (4) | 0·60 (1) | 1·44 (2) | 1·02 (1) | — |
| Ambikapur | — | — | — | — | — | 1·50 (1) | 1·92 (6) | 1·74 (4) | 0·48 (1) | — | — |

and thick precipitation. Relatively higher amounts of iodide were found in the rain water collected from industrial cities (Raipur, Bhilai, Korba and Bilaspur) and this was due to industrial emission.

### Conclusion

The present method is simple, fast, sensitive and selective and is suitable for characterization of iodide at trace levels in water samples (rain, surface and ground). The use of the surfactant, CPC, increases by more than the three-fold sensitivity of the conventional FIA method based on the catalytic destruction of Fe(III)–$SCN^-$ complex with $NO_2^-$. In terms of its simplicity and high sample throughput, this method seems to be superior to most of methods reported for the automatic analysis of iodide in water samples.

### Acknowledgement

The authors are grateful to UGC, New Delhi, DST, New Delhi and the Alexander von Humboldt Foundation, Bonn for financial assistance towards this work.

### References

1. Guyton, C., *Textbook of Medical Physiology* (W. B. Sanders, London, 1986).
2. Matthes, W., Flucht, R., and Stoeppler, M., *Fresenius Z. Analytical Chemistry*, **291** (1978), 217.
3. Matthews, A. D. and Riley, J. P., *Analytica Chimica Acta*, **51** (1970), 295.
4. Babko, A. K. and Pilipenko, A. T., *Photometric Analysis: Method of Determining Non-Metals* (Mir Publishers, Moscow, 1976).
5. Chandrawanshi, S. and Patel, K. S., *Fresenius Z. Analytical Chemistry*, **352** (1995), 599.
6. Malmstadt, H. V. and Hadjiioannou, T. P., *Analytical Chemistry*, **35** (1963), 2157.
7. Toledano, M., Gutierrez, M. C., Gomez-Hens, A., and Perez-Hens, D., *Analusis*, **17** (1989), 514.
8. Moxon, R. D. and Dixon, E. J., *Analyst*, **105** (1980), 344.
9. Moxon, R. E., *Analyst*, **109** (1984), 425.
10. Oguma, K., Kitada, K., and Kuroda, R., *Microchimica Acta*, **110** (1993), 71.
11. Nakayama, E., Kimoto, T., Isshiki, K., Sohrin, Y., and Okazaki, S., *Marine Chemistry*, **27** (1989), 105.
12. Luther, L. W., Swartz, C. B., and Ullman, W. J., *Analytical Chemistry*, **60** (1988), 1721.
13. Han, K., Koch, W. F., and Pratt, K. W., *Analytical Chemistry*, **59** (1987), 731.
14. Ito, K. and Sunahara, H. J., *Chromatography*, **502** (1990), 121.
15. McTaggart, A. R., Butler, E. C. V., Haddad, P. R., and Middleton, J. H., *Marine Chemistry*, **47** (1994), 159.
16. Tripathi, A. N., Chikhalikar, S., and Patel, K. S., *International Journal of Automatic Chemistry*, submitted.

